# Effectiveness of Super Floss and Water Flosser in Plaque Removal for Patients Undergoing Orthodontic Treatment: A Randomized Controlled Trial

**DOI:** 10.1155/2022/1344258

**Published:** 2022-08-31

**Authors:** Nozha Sawan, Afnan Ben Gassem, Faisal Alkhayyal, Aroob Albakri, Nada Al-Muhareb, Eman Alsagob

**Affiliations:** ^1^Preventive Dental Sciences Department, College of Dentistry, Princess Nourah Bint Abdulrahman University, Riyadh, Saudi Arabia; ^2^Department of Pediatric Dentistry and Orthodontics, College of Dentistry, Taibah University, Medina, Saudi Arabia; ^3^Director of the Quality & Patient's Safety Department at Riyadh Specialized Dental Center, Riyadh, Saudi Arabia; ^4^College of Dentistry, Princess Nourah Bint Abdulrahman University, Riyadh, Saudi Arabia

## Abstract

**Objectives:**

This study aimed to compare the effectiveness of super floss and water flosser in plaque removal for patients undergoing orthodontic treatment.

**Methods:**

A single-blind, randomized, controlled, parallel clinical trial with a split-mouth protocol was conducted on young adult orthodontic patients who were recruited from Riyadh Specialized Dental Center in Riyadh, Saudi Arabia. The type of floss used was randomly assigned to each side of the oral cavity; Super-Floss® (Oral-B) was used on one side, while the Waterpik® water flosser was used on the other. Patients' plaque level was assessed using Rustogi et al. modified navy plaque index (RMNPI) at baseline and immediately after cleaning.

**Results:**

A total of 62 subjects were screened; however, only 34 subjects were enrolled in the study with an equal number of males and females. Overall, the plaque score was significantly reduced from 0.56 ± 0.35 to 0.13 ± 0.26 in the super floss group and from 0.61 ± 0.35 to 0.13 ± 0.28 in the water flosser group. There was no significant difference between the mean difference of super floss and water flosser (*p*=0.951). On the other hand, there was no significant difference between both groups in terms of the preintervention plaque score (*p*=0.379). The water flosser had a greater effect size on plaque removal compared to super floss on distal interproximal surface of the molar tooth with a mean difference of (−0.21, 95% CI: 00.37 to −0.04, *p*=0.033).

**Conclusions:**

The use of super floss or water flosser as interproximal aids for plaque removal in patients undergoing orthodontic treatment are both effective. Trial registration. ISRCTN, ISRCTN83875016. Registered 12 September 2021-retrospectively registered, https://www.isrctn.com/ISRCTN83875016.

## 1. Introduction

Patients who wear fixed orthodontic appliances must maintain high standards of oral hygiene; otherwise, they will experience a range of complications, including decay, enamel demineralization, gingivitis, gingival hyperplasia, and periodontitis [1]. Multiple studies demonstrated that using fixed orthodontic appliances can make it more challenging to maintain good oral hygiene due to the plaque accumulation around the archwires, bands, and brackets [2–4]. The fixed appliance can also change the bacterial composition of the oral environment, enable bacterial plaque retention, reduce self-cleaning capabilities, and trigger gingival infection or enamel decalcification and white spot lesions with soft-tissue recession and teeth abrasion [5]. The current evidence suggests that daily patient-administered mechanical plaque control should be considered the gold standard, and the most important factor in the management and reduction of plaque accumulation, even if professional oral hygiene therapy is necessary to remove subgingival plaque and reduce gingival inflammation [6, 7]. For this reason, several articles advocate using a combination of toothbrush (electric or sonic) and dental flosser (wire or water jet). Oral hygiene and plaque index were improved by both an electric toothbrush and a sonic toothbrush, without damaging soft or hard tissues, according to a recent systematic review [8]. The primary objective of any oral hygiene program should be to ensure that patients are motivated to pursue good oral hygiene and that they remain compliant with their oral hygiene program throughout the treatment period [9].

Various devices are available to help orthodontic patients maintain good oral hygiene, including essential manual toothbrushes and toothpaste, electronic toothbrush, dental floss, brushes for interproximal hygiene, and oral irrigators, such as dental water floss [10]. According to the American Dental Association (ADA), water flossers have been tested to be safe and effective at removing plaques, which are associated with a higher risk for cavities and gum disease. In addition, a water flosser can reduce gingivitis, the early form of gum disease [11]. One of the main challenges practitioners often encounter is that orthodontic patients, most of whom are young, cannot be relied upon to engage in preventative health behavior. Furthermore, changing a patient's oral hygiene habits might be difficult. This situation can be exacerbated because orthodontic appliances can make it more challenging to maintain good oral hygiene [12]. For instance, the use of a string-waxed floss for interdental cleaning relies on special floss or a threading device; however, it can be time-intensive and challenging. Some studies have demonstrated that super floss achieves superior outcomes to regular waxed floss and can enhance gingival health [13]. Super floss consists of three primary elements: a strengthened-end dental floss threader that makes it easier to position the floss under the orthodontic wires, a fuzzy floss that can clean around any wide gaps, and orthodontic brackets, and standard dental floss that can eliminate plaque from under the gingival contours [14]. Electric devices, such as water flossers, have also been made available to help orthodontic patients overcome some of the issues associated with the use of standard string floss while also achieving the same degree of effectiveness [15].

For instance, a research study by Sharma et al. [16] found that using a water flosser in combination with manual brushing decreased bleeding on probing scores by 41.2% over a period of 28 days. The pulsing action of the water flosser compresses and decompresses the gingival tissue. This enables the water to the subgingival and interdental regions surrounding the tooth to remove plaque, bacteria, and debris, especially unreachable regions, by standard toothbrushes [17]. Although several clinical studies have proven the benefit of water floss in reducing gingival inflammation, bleeding, and pathogenic bacteria, most of these studies focused on non-orthodontic patients such as patients with periodontal disease, patients with implants, crowns, or bridges, and patients with diabetes [10, 17–21]. Studies investigating the efficacy of dental water floss on oral hygiene control of orthodontic patients are limited, and its impact on reducing supragingival plaque biofilm remains unclear [22]. This determines the need for studying the effect of this device on an orthodontic patient sample in particular and whether it is superior or as effective compared to super floss. Hence, this randomized control trial aimed to evaluate the effectiveness of super floss (Oral-B Super Floss) and water flosser (Waterpik Cordless Freedom Water Flosser) in plaque removal in orthodontic patients.

## 2. Methods

### 2.1. Study Design

In this single-blind, randomized, controlled, parallel clinical trial with a split-mouth protocol, we followed the CONSORT Statement for reporting randomized trials [23]. Verbal and written consents were obtained from all included patients. Young adult orthodontic patients were recruited and randomly selected with an allocation ratio of 1 : 1 from Riyadh Specialized Dental Center in Riyadh, Saudi Arabia. The study was reviewed and exempted by the Institutional Review Board (IRB) of Princess Nourah bint Abdulrahman University (18-0241). In addition, investigators underwent a bioethics-training course from the National Committee of BioEthics (NCBE) in Saudi Arabia. The protocol of this study has been registered in the ISRCTN registry (ISRCTN83875016).

### 2.2. Inclusion and Exclusion Criteria

Patients were recruited if they were male or female between 18 and 35 years old who approached the end of their orthodontic treatment. Patients who had braces from the right first molar to the left first molar with pocket depth ≤3 mm and had not used any floss type for the last 24 hours were included. Patients with systemic diseases, craniofacial anomalies, periodontal problems, spacing, or missing teeth in the examined arch, and those who were smokers were excluded from the study.

### 2.3. Examiners' Calibration

**Figure 1:**
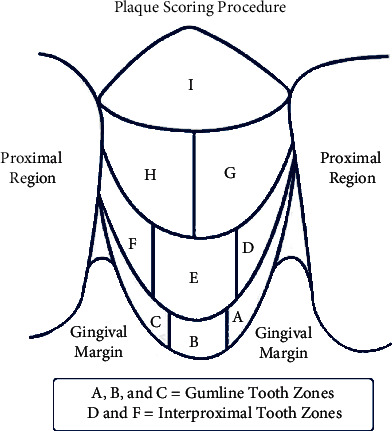
Plaque scoring procedure; A, B, and C; gumline tooth zones, D and F; interproximal tooth zones.

The two examiners were calibrated-each independently examined four patients using Rustogi et al. [24], modified navy plaque index (RMNPI) (see Figure 1). The observed agreement with Cohen's kappa statistic revealed a value of 0.8.

### 2.4. Intervention

In a single visit, the split-mouth technique was performed to compare consistency in both groups. In addition, RMNPI was adapted to measure plaque levels of all subjects at baseline with the use of the WHO probe [24]. The RMNPI was chosen because it allows for the recording of scores and the calculation of the mean plaque index before and after oral hygiene application. A separate researcher delivered standardized oral hygiene instructions to all subjects, using the modified bass technique and a standard toothbrush (soft-bristled brush with fluoridated toothpaste) and explained to the patients the correct method of using interdental cleaning techniques manufacturer's instructions. The type of floss used was randomly assigned to each side of the oral cavity; Super Floss® (Oral-B) was used on one side, while the Waterpik® water flosser was used on the other. The tip of the flosser is held close at a 90-degree angle to the tooth at the gingival margin and follows a pattern around the mouth to clean all facial and lingual areas of the teeth. The pressure of this Waterpik range between 45 and 75 PSI (3.103 to 5.171 Bar), with a flow rate per minute of 8 ounces (237 ml), and 1200 pulses per minute. All participants had two minutes to brush their teeth and another two minutes to clean their interproximal teeth.

### 2.5. Outcome Measurement

The plaque index of each side was taken and compared with the baseline score. Examiners who recorded the plaque index before and after the trial were blinded regarding the type of floss used for each side of the mouth. Respectively, a canine, one premolar, and one molar were selected for evaluation. There are nine sections to score with the RMNPI (Figure 1). Sections are then combined to provide data for the marginal and proximal regions. Plaque is assessed for each tooth area and is scored using the following scale: 0 = absent and 1 = present. This study focuses on areas that can be cleaned with floss; mean plaque index (MPI) was scored only for proximal areas (A, D, C, and F). However, due to orthodontic brackets, surfaces (G and H) were not recorded and the number of surfaces was modified correspondingly.

### 2.6. Sample Size Calculation

Using the G*∗*Power 3.1.9.4 software, the sample size was calculated. Based on an effect size of 1 [18], alpha of 5%, and a power of 80%; the minimum expected sample was found to be thirty-four subjects. The effect size was acquired by calculating the average of the mean difference between the experimental and control groups in terms of the efficacy in improving the plaque index (in whole mouth, approximal, marginal, facial, and lingual) [18, 25].

### 2.7. Statistical Analysis

The collected data were entered from the paper-based records into SPSS version 22. Data have been tested in terms of normality using the Kolmogorov–Smirnov test and the Shapiro–Wilk test. Parametric continuous data were presented as mean and standard deviation (SD). Categorical data were presented as frequency and percentage. Data were analyzed using paired *t*-test to compare the plaque scores of each interdental aid (before and after) and to compare the effectiveness of the two interdental aids in plaque removal from the different teeth (canine, premolar, and molar) and the different surface areas (mesial and distal). A *p* value of less than 0.05 was considered significant.

## 3. Results

### 3.1. Demographic Characteristics

**Figure 2:**
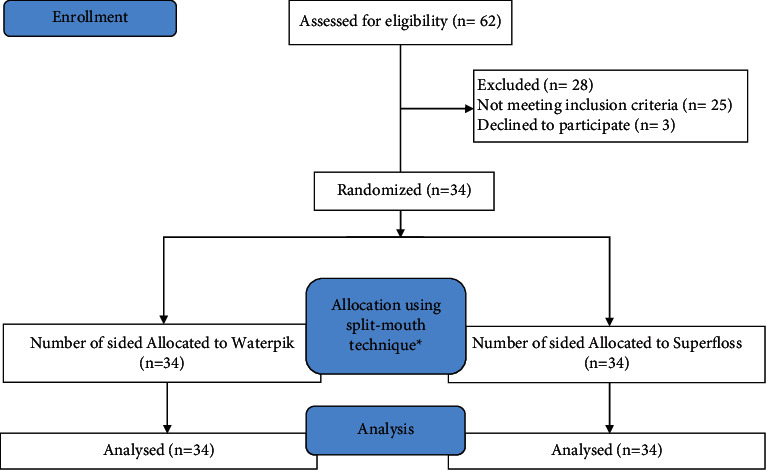
Consort diagram, participants' flow chart and inclusion for per protocol analysis.

A total of 62 subjects were screened; however, only 34 subjects were enrolled in the study with an equal number of males and females (Figure 2). The mean age of included participants was 23.7 ± 7.7 years. Both genders were equally represented in this study 1 : 1.

### 3.2. Plaque Score

Overall, the plaque score was significantly (<0.001) reduced from 0.56 ± 0.35 to 0.13 ± 0.26 in the super floss group and from 0.61 ± 0.35 to 0.13 ± 0.28 in the water flosser group. There was no significant difference between the mean difference of super floss and water flosser groups (*p*=0.951). According to the type of tooth, both groups showed a significant (*p* < 0.001) reduction in the plaque score postintervention in canine, premolars, and molars. However, the mean difference was higher in the water flosser group compared with the super floss in all teeth, with no statistically significant difference.  Table 1 displays the effectiveness of the two techniques for plaque removal from the different teeth.

Table 2 demonstrates that the water flosser and super floss had a comparable effect size in terms of plaque removal in both mesial and distal interproximal surfaces of all teeth, except for distal molar where the water flosser was more effective in reducing the plaque score with a mean difference of (−0.21, 95% CI: 00.37 to −0.04, *p*=0.033). Based on a split-mouth method, our analysis showed that both interventions significantly reduced the plaque score in both left and right sides with no significant difference between super floss and water flosser groups (Table 3).

## 4. Discussion

Orthodontic treatment with fixed appliances is considered a risk factor for plaque accumulation due to the difficulty of following proper brushing techniques and access limitations [26, 27]. Thus, effective homecare regimens directed toward the unique challenges of patients undergoing fixed or other orthodontic appliances are always emphasized in orthodontic treatment to meet acceptable standards as this will positively impact the treatment results. However, as mentioned previously, the literature on the use of water dental jets on orthodontic patients is limited and the majority of studies available are conducted on non-orthodontic patients. Hence, the purpose of this study was to determine the effectiveness of a water flosser on plaque removal in orthodontic patients who are most susceptible to plaque accumulation compared to a more traditional interdental cleaning technique, namely, the super floss.

The current study revealed that both water flosser and super floss effectively reduced plaque levels, showing significantly lower postcleaning plaque scores than precleaning scores. However, when the results of the two groups were compared, it was found that water flosser was comparable to super floss in almost all teeth, surfaces, and mouth sides; however, it was more effective than super floss at reducing plaque score in distal molar tooth. Although these findings are in agreement with a study that demonstrated that the performance of dental water jets was comparable to the dental floss cleaning aid with no differences detected among the groups [28], many previous studies reported the superiority of dental water jets in removing interproximal plaque at 2 and 4 weeks interval [29–31].

In the single-blind randomized control trial, Sharma and his colleagues compared between air floss and water floss in terms of the reduction of gingivitis. They reported that both groups significantly reduced the gingivitis, bleeding on probing, and plaque from all regions and time points measured (*p* < 0.001). They also demonstrated that water floss was more effective than air floss in terms of reducing plaque and gingivitis in all areas measured (*p* < 0.001). Therefore, they concluded that water flosser was significantly more effective than air floss for reducing gingivitis and plaque [16]. In addition, Goyal et al. reported that the water flosser was more effective than super floss in terms of gingivitis and bleeding [18]. However, other studies reported no significant differences between dental water jets and dental floss in overall plaque scores but did not breakdown the comparison to the interproximal surfaces, which may be a reason for the contradictory result. Had they compared plaque levels on interproximal surfaces alone, the results may be different (and more relevant), especially when comparing interdental cleaning aids that are designed to remove plaque more specifically from the interproximal dental surfaces.

Mazzoleni et al. compared the efficacy of dental water jets combined with tooth brushing to tooth brushing alone at baseline, one, three, and six months intervals [32].

The only significant difference in plaque-level reduction was on the molar at the 6-month follow-up for the dental water jet group and on the premolar at the 6-month follow-up for the control group. Although this is an encouraging finding that water flosser may be beneficial in removing plaque from hard to reach areas, i.e., proximal surfaces that are more distally located, it may be just the effect of irrigation reducing the thickness of plaque, which may sometimes be undetectable using 2-dimensional scoring systems [33]. Recently, many authors proposed the use of the water jet with ozonated water, in order to reduce the bacterial load and accelerate the healing processes. Butera and his colleagues conducted a randomized control trial that revealed a significant improvement in the mean probing pocket depth, plaque index, bleeding score, and bleeding on probing [34].

The results of the study should be interpreted with caution as the plaque index was measured directly after cleaning only and the prolonged effect of dental water jets was not measured. The purpose of the study was to determine the instant effects of the interdental aids on plaque removal, specifically as interproximal areas are where plaque mostly accumulates and is the site where infection and gingivitis are likely first to occur. This may have introduced a Hawthorne effect where the study subjects realized they were in a study and being observed, and hence, their cleaning performance was influenced by this effect. Future studies could focus on the long-term effects of dental water jets on orthodontic patients and include other outcomes such as bleeding index, which can only be measured longitudinally, reducing the probability of biased results. Studies of longer duration will also more clearly demonstrate which method is more convenient for the participants after using super floss and water flosser. In addition, a larger sample size would improve the power of the study; however, this may be challenging to achieve as the study was single-centered. A multicentered study would allow recruiting a larger number of patients, resulting in detecting a greater difference between the two groups. Moreover, another significant limitation is the difficulty to obtain a complete standardization among the two interventions.

## 5. Conclusion

In conclusion, the use of super floss or water flosser as interproximal aid for plaque removal in patients undergoing orthodontic treatment is equally effective in reducing plaque levels, and hence, both interproximal tools proved to be safe and efficient for use by orthodontic patients. Further studies are required to investigate the impact of daily use of the combination of toothbrushes and dental floss, and the role of using the water jet with ozonated water on oral hygiene, plaque index, and bleeding score.

## Figures and Tables

**Table 1:** Comparison of plaque levels between super floss and water floss pre- and post-cleaning on selected teeth.

	Super floss			Water flosser			Super floss	Water flosser
	Pre-intervention	Post-intervention	Pre-intervention	Post-intervention	Pre-intervention	Post-intervention		
Canine mean ± SD 95% CI	0.57 ± 0.43 0.42–0.72	0.13 ± 0.26 0.04–0.22	**<0.001**	0.60 ± 0.40 0.45–0.74	0.13 ± 0.28 0.03 ± 0.22	**<0.001**	−0.007 (−0.13 to 0.12)	0.726
Molar mean ± SD 95% CI	0.53 ± 0.48 0.36–0.70	0.15 ± 0.31 0.04–0.26	**<0.001**	0.61 ± 0.47 0.45–0.77	0.07 ± 0.17 0.01–0.13	**<0.001**	−0.08 (−0.19 to 0.03)	0.173
Premolar mean ± SD 95% CI	0.60 ± 0.45 0.44–0.75	0.18 ± 0.30 0.08–0.29	**<0.001**	0.63 ± 0.43 0.48–0.78	0.095 ± 0.20 0.027–0.16	**<0.001**	−0.09 (−0.21 to 0.03)	0.206

A *p* value of less than 0.05 was considered significant.

**Table 2:** Comparison between super floss and water floss pre- and post-cleaning for mesial and distal sides of selected teeth.

Segment	Tooth	Super floss			Water flosser			Super floss	Water flosser
		Pre-intervention	Post-intervention	Pre-intervention	Post-intervention	Pre-intervention	Post-intervention		
Mesial	Canine mean ± SD 95% CI	0.61 ± 0.49 0.45–0.79	0.12 ± 0.33 0.004–0.23	**<0.001**	0.62 ± 0.49 0.45–0.79	0.12 ± 0.33 0.004 ± 0.23	**<0.001**	0.00 (−0.17 to 0.17)	1.00
	Molar mean ± SD 95% CI	0.53 ± 0.51 0.36–0.70	0.09 ± 0.29 0.01–0.18	**<0.001**	0.61 ± 0.49 0.45–0.79	0.09 ± 0.29 0.01–0.18	**<0.001**	0.00 (−0.15 to 0.15)	0.513
	Premolar mean ± SD95% CI	0.65 ± 0.49 0.47–0.82	0.13 ± 0.35 0.004–0.26	**<0.001**	0.73 ± 0.45 0.57–0.89	0.14 ± 0.35 0.004–0.27	**<0.001**	0.00 (−0.17 to 0.17)	0.491

Distal	Canine mean ± SD 95% CI	0.50 ± 0.51 0.32–0.68	0.15 ± 0.36 0.02–0.27	**0.001**	0.56 ± 0.50 0.38–0.73	0.12 ± 0.33 0.004 ± 0.23	**<0.001**	−0.03 (−0.19 to 0.13)	0.513
	Molar mean ± SD 95% CI	0.53 ± 0.51 0.35–0.71	0.24 ± 0.43 0.09–0.39	**0.004**	0.62 ± 0.49 0.45–0.79	0.03 ± 0.17 0.00–0.09	**<0.001**	−**0.21** (−**0.37** to −**0.04**)	**0.033**
	Premolar mean ± SD 95% CI	0.53 ± 0.51 0.35–0.71	0.24 ± 0.43 0.09–0.39	**0.002**	0.56 ± 0.51 0.38–0.73	0.09 ± 0.29 0.01–0.19	**<0.001**	−0.15 (−0.34 to 0.05)	0.134

**Table 3:** Comparison of plaque levels between super floss and water floss pre- and post-cleaning on selected teeth on both left and right sides.

		Super floss			Water flosser			Mean difference	*p*-value
		Pre-intervention	Post-intervention	*p*-value	Pre-intervention	Post-intervention	*p*-value		
All teeth	Left	0.44 ± 0.26	0.11 ± 0.13	**0.001**	0.66 ± 0.32	0.17 ± 0.22	**<0.001**	0.062 (−0.10 to 0.22)	0.482
	Right	0.66 ± 0.32	0.17 ± 0.22	**<0.001**	0.47 ± 0.25	0.11 ± 0.13	**0.001**	−0.061 (−0.22 to 0.10)	0.783

Canine	Left	0.46 ± 0.41	0.11 ± 0.29	**0.015**	0.69 ± 0.36	0.20 ± 0.31	**0.002**	0.09 (−0.07 to 0.25)	0.258
	Right	0.64 ± 0.45	0.19 ± 0.25	**0.002**	0.47 ± 0.44	0.07 ± 0.26	**0.010**	−0.10 (−0.31 to 0.11)	0.317

Molar	Left	0.47 ± 0.44	0.23 ± 0.37	**0.038**	0.74 ± 0.42	0.08 ± 0.17	**<0.001**	−0.13 (−0.37 to 0.11)	0.196
	Right	0.58 ± 0.51	0.09 ± 0.25	**0.003**	0.45 ± 0.48	0.07 ± 0.18	**0.010**	0.017 (−0.12 to 0.15)	0.705

Premolar	Left	0.53 ± 0.44	0.20 ± 0.32	**0.004**	0.82 ± 0.34	0.14 ± 0.22	**<0.001**	−0.017 (−0.28 to 0.24)	0.989
	Right	0.64 ± 0.47	0.17 ± 0.29	**0.002**	0.40 ± 0.43	0.03 ± 0.13	**0.015**	−0.15 (−0.34 to 0.04)	0.131

## Data Availability

The datasets used and/or analyzed during the current study are available from the corresponding author on reasonable request.

## Abbreviations

RMNPI: Rustogi et al. modified navy plaque index
IRB: Institutional Review Board
NCBE: National Committee of Bioethics
WHO: World Health Organization
MPI: Mean plaque index
SPSS: Statistical Program for Social Sciences
SD: Standard deviation.

## Supplementary Materials

Supplementary Materials Supplementary file 1 shows the CONSORT cheklist for reporting randomized trials.
